# Assessment of the Effects of Aerobic Fitness on Cerebrovascular Function in Young Adults Using Multiple Inversion Time Arterial Spin Labeling MRI

**DOI:** 10.3389/fphys.2020.00360

**Published:** 2020-04-21

**Authors:** Catherine Foster, Jessica J. Steventon, Daniel Helme, Valentina Tomassini, Richard G. Wise

**Affiliations:** ^1^Cardiff University Brain Research Imaging Centre (CUBRIC), School of Psychology, Cardiff University, Cardiff, United Kingdom; ^2^Cardiff University Brain Research Imaging Centre (CUBRIC), School of Physics and Astronomy, Cardiff University, Cardiff, United Kingdom; ^3^Neuroscience and Mental Health Research Institute (NMHRI), School of Medicine, Cardiff University, Cardiff, United Kingdom; ^4^Department of Anaesthetics and Intensive Care Medicine, Cardiff University School of Medicine, Cardiff, United Kingdom; ^5^Division of Psychological Medicine and Clinical Neurosciences, School of Medicine, Cardiff University, Cardiff, United Kingdom; ^6^Department of Neuroscience, Imaging and Clinical Sciences, “G. D’Annunzio University” of Chieti-Pescara, Chieti, Italy; ^7^Institute for Advanced Biomedical Technologies (ITAB), “G. D’Annunzio University” of Chieti-Pescara, Chieti, Italy

**Keywords:** arterial spin labeling, cerebral blood flow, cerebral hemodynamics, cerebrovascular reactivity, exercise

## Abstract

This cross-sectional study investigated the effects of aerobic fitness on cerebrovascular function in the healthy brain. Gray matter cerebral blood flow (CBF) and cerebrovascular reactivity (CVR) were quantified in a sample of young adults within a normal fitness range. Based on existing Transcranial Doppler ultrasound and fMRI evidence, we predicted a positive relationship between fitness and resting gray matter CBF and CVR. Exploratory hypotheses that higher $\dot{V}$O_2_peak would be associated with higher GM volume and cognitive performance were also investigated. 20 adults underwent a $\dot{V}$O_2_peak test and a battery of cognitive tests. All subjects also underwent an MRI scan where multiple inversion time (MTI) pulsed arterial spin labeling (PASL) was used to quantify resting CBF and CVR to 5% CO_2_. Region of interest analysis showed a non-significant inverse correlation between whole-brain gray matter CBF and $\dot{V}$O_2_peak; *r* = −0.4, *p* = 0.08, corrected *p* (*p*′) = 0.16 and a significant positive correlation between $\dot{V}$O_2_peak and whole-brain averaged gray matter CVR; *r* = 0.62, *p* = 0.003, *p*′ = 0.006. Voxel-wise analysis revealed a significant inverse association between $\dot{V}$O_2_peak and resting CBF in the left and right thalamus, brainstem, right lateral occipital cortex, left intra-calcarine cortex and cerebellum. The results of this study suggest that aerobic fitness is associated with lower baseline CBF and greater CVR in young adults.

## Introduction

Aerobic fitness has emerged as a modifiable lifestyle factor which reduces the risk of all-cause mortality (Kodama et al., 2009; Lee et al., 2010), cardiovascular events (Haskell et al., 2007; Lee et al., 2010; Roque et al., 2013) and protects the brain against age and disease-related decline (Hillman et al., 2008; Hayes et al., 2014a; Nishijima et al., 2016). In addition, brain structure and function are closely linked; to maintain healthy brain tissue, adequate energy must be supplied. Many studies have investigated the link between aerobic fitness and brain structure (Voss et al., 2015) but the relationship between aerobic fitness and cerebrovascular function has not been extensively studied in humans. The present study focuses on how aerobic fitness may affect the cerebrovascular processes which maintain delivery of brain nutrients.

Neuroimaging studies have provided evidence of a neuroprotective effect of aerobic fitness in older adults, linking higher aerobic fitness with greater brain volume (Erickson et al., 2011) and cerebral blood flow (CBF) (Thomas et al., 2013). Aerobic fitness has also been associated with greater cerebrovascular reactivity (CVR), which reflects the ability of the cerebral vessels to dilate, or the vascular reserve, assessed using transcranial Doppler (TCD) ultrasound (Bailey et al., 2013) in both older and younger adults. However, Barnes et al. (2013), using the same technique, did not find a relationship between CVR and $\dot{V}$O2_max_ in younger adults despite observing the same trend as Bailey et al. (2013) in older adults. Further, the findings of Bailey et al. (2013) and Barnes et al. (2013) in older adults, do not agree with the MRI results of Thomas et al. (2013) who reported a lower BOLD-based CVR in masters athletes vs. sedentary young and age-matched controls. Finally, Gauthier et al. (2015) found that in older adults, BOLD CVR was negatively correlated with $\dot{V}$O_2_max in frontal regions but positively correlated in periventricular white matter and portions of the somatosensory cortex. These conflicting CVR data represent an issue of inconsistent findings regarding aerobic fitness and its effects on cerebrovascular function across the lifespan.

Recent findings from Hwang et al. (2017) using TCD, also suggest that young adults with higher levels of aerobic fitness have a greater cerebrovascular response to hypercapnia in the middle cerebral artery (MCA), during exercise, but found no differences at rest. The fitter group also scored better on tests of fluid reasoning, but not on any other tasks included in the cognitive battery. However, as we are interested in how aerobic fitness may lead to general improvements in cerebrovascular health rather than vascular regulation during exercise, the current cross-sectional study focused on the relationship between aerobic fitness and cerebrovascular function in the resting brain of young, healthy adults. As the TCD literature suggests a positive relationship between fitness and CVR in young people, it is important to establish whether functional MRI reports the same effects, as TCD reflects the velocity of arterial blood rather than perfusion of gray matter.

The objective of this study was to quantify using pulsed arterial spin labeling (PASL) MRI at multiple post-labeling delay times, variations in resting CBF and CVR, two widely used indices of cerebrovascular function, in a sample of young, healthy adults within a normal range of fitness levels (i.e., sedentary and active, but not athletes in structured training). Based on the extant TCD and fMRI evidence, we predicted a positive relationship between fitness and resting gray matter CBF. We also expected to see a positive correlation between CVR to CO_2_ and $\dot{V}$O_2_peak, a measure of aerobic fitness, in line with previous findings (Bailey et al., 2013; Hwang et al., 2017). Tests chosen to measure a number of cognitive domains were included to examine the exploratory hypotheses that higher $\dot{V}$O_2_peak would be associated with better cognitive performance and higher gray matter volume. If CBF and CVR differences due to fitness were evident, then demonstrating additional cognitive benefits would provide evidence for functionally relevant effects of neurobiological differences.

## Materials And METHODS

### Participants

Twenty healthy adults (11 females, mean age 25 ± 4.6) were recruited from Cardiff University. The study was approved by the Cardiff University School of Psychology Research Ethics Committee and performed in accordance with the guidelines stated in the Cardiff University Research Framework (version 4.0, 2010). All participants were non-smokers and educated to university level. Informed written consent was obtained from all subjects.

### Study Procedures

The study consisted of 3 lab visits. In Visit 1, eligibility screening for MRI, respiratory modulations (see Supplementary Figure S1) and intensive exercise was carried out. Contraindications to exercise were assessed using the Physical Activity Readiness Questionnaire (PARQ). Sociodemographic information was recorded and estimations of weekly activity level were established using the International Physical Activity Questionnaire (IPAQ) Short Form (Craig et al., 2003). Elevated CO_2_ inhalation can cause sensations of breathlessness, light headedness and anxiety in some individuals. For this reason, all volunteers took part in a gas modulation session in a MR scanner simulator. A stepwise protocol was employed to allow participants time to become accustomed to CO_2_ inhalation (see Supplementary Figure S1).

In Visit 2, volunteers completed 7 cognitive tests, administered by the same researcher for all participants, prior to the fitness test. The tests, covering a range of cognitive domains, were chosen as they are validated for use in patient and control samples (see Table 1).

**TABLE 1:** List of cognitive tests used in this study and domains they are intended to assess.

Test	Domain measured
Speed and capacity of language processing (SCOLP) (Baddeley et al., 1992)	Information processing, speed, and language comprehension
Forward digit span	Working memory
Letter fluency (categories)	Verbal fluency
Conners continuous performance test (Conners, 2000)	Sustained attention and response inhibition
Trail making test (part B)	Processing speed and executive function
Symbol digit modalities test	Information processing speed

### Fitness Test

The fitness test was also performed in Visit 2. The PAR-Q was conducted a second time to identify potential risk factors associated with exercise in the unlikely case of circumstance changes from Visit 1. The $\dot{V}$O_2_peak test protocol used has previously been described by Collett et al. (2011) and Cooney et al. (2013). The test began with a 2 min unloaded warm up at 50 revolutions per minute (rpm) on a Lode cycle ergometer (Lode, Groningen, Netherlands). During the test, participants maintained a constant 50 rpm and work rate was increased from 50 watts by 25 watts every 2 min. The test was terminated if any of the following criteria were reached; work rate fell below 45 rpm for >10 s, volitional exhaustion occurred, or maximal predicted heart rate exceeded 100%. At the end of each 2 min step Borg ratings of perceived exertion, on the CR10 scale (Borg and Kaijser, 2006) were used to record perceived heaviness of legs and breathing.

Blood lactate concentration was sampled from the earlobe using Unistik 3 1.8 mm lancets (Williams Medical, Caerphilly, United Kingdom) and tested using the Lactate Plus system (Nova Biomedical, Waltam, MA, United States) at baseline, 2 min intervals and at exhaustion. Blood pressure (BP) was recorded at baseline, HR at baseline and 2 min intervals during the test. Respiratory gas exchanges were continuously monitored using breath by breath analysis with the Cortex Metalyser 3b (Cortex Biophysik Metalyzer, Germany). $\dot{V}$O_2_peak was taken as the highest value over a 30 s block average at the maximum recorded work rate. Heart rate (HR) and work rate were also recorded at baseline and at 2 min intervals and electrocardiography (ECG) was monitored for the duration of the test. Following termination of the $\dot{V}$O_2_peak test participants were monitored for 10 min. In this 10 min recovery period blood pressure, HR and blood lactate were sampled every 2 min before subjects left the lab.

### MRI Acquisition

In Visit 3, volunteers underwent the MR scan. Images were acquired on a 3T whole body MRI system (GE Excite HDx, Milwaukee, WI, United States) using an eight-channel receive-only head coil. Heart rate was recorded using a pulse oximeter and a respiratory bellows monitored breathing. A sampling line connected to the face mask of the breathing circuit (Tancredi et al., 2014) was used to monitor the partial pressure of end-tidal CO_2_ (P_ET_CO_2_) and O_2_ (P_ET_O_2_) via the Biopac < *cps*:*sup* > ® < /*cps*:*sup* > system (Biopac, Worcestershire, United Kingdom). The MEDRAD system (MEDRAD, Pittsburgh, PA, United States) was used to monitor O_2_ saturation throughout the experiment.

PASL data were acquired using a single subtraction PICORE QUIPSS II (Wong et al., 1998) with a dual-echo gradient-echo readout (Liu et al., 2002) and spiral k-space acquisition (Glover and Lai, 1998) the first echo being used for CBF quantification. Data were acquired at 8 inversion times; 400, 500, 600, 700, 1100, 1400, 1700, and 2000 ms. QUIPSS II cut-off at 700 ms meant that short and long inversion times were acquired in separate runs. 16 and 8 tag-control pairs were acquired for each of the short and long inversion time respectively. A variable TR was used for efficiency, 1200–1500 ms for short TI data (400–700 ms) and 1700–2600 ms for long TIs (1100–2000 ms). Other acquisition parameters were; TE = 2.7 ms, voxel size = 3.1 × 3.1 × 7 mm^3^, matrix size 64 × 64 mm, FOV = 19.8 cm, flip angle = 90°, inter-slice time 55 ms, 15 slices, slice gap 1.5 mm for maximum brain coverage. Label thickness was 200 mm with 10 mm gap between the end of the label and the most proximal imaging slice.

A calibration image without any labeling was acquired before the perfusion mages using the same acquisition parameters, except a long TR; this was used to obtain the equilibrium magnetization of cerebrospinal fluid (M_0_, CSF), needed for the quantification of CBF. The acquisition parameters for this CSF were identical to the PASL acquisition, except that no labeling was applied so that the image was acquired with fully relaxed magnetization. A minimal contrast image was also acquired to correct for coil inhomogeneities with TE = 11 ms, TR = 2 s. The PASL acquisition was performed twice, first during rest, then while volunteers were hypercapnic. The hypercapnic modulation was carried out using the prospective control method described by Tancredi and Hoge (2013). A gas mixing chamber constructed in-house had three feeding lines coming in for the delivery of medical air, 5% CO_2_, and medical oxygen, the latter incorporated as a safety backup but not used during experimentation, the circuit is described in detail by Tancredi et al. (2014). P_ET_CO_2_ elevation of 7mmHg was targeted using 5% CO_2_ (balance air) and the scan commenced once participants reached the target CO_2_ level. ASL data acquisition lasted ∼6 min during rest, followed by a delay to allow the onset of hypercapnia. Once a P_ET_CO_2_ elevation of approximately 7mmHg was achieved, the ∼6 min ASL acquisition was repeated.

A T1-weighted 3D structural fast spoiled gradient echo (FSPGR) scan was also acquired for registration of functional data and voxel-based morphometry (VBM) analysis; TR/TE = 7.8/2.9 ms, resolution = 1 mm isotropic.

### Data Analysis

#### $\dot{V}$O_2_peak Calculation

$\dot{V}$O_2_peak was calculated from the respiratory gas exchanges which were continuously monitored using breath-by-breath analysis and averaged over 10 s blocks. $\dot{V}$O_2_peak was defined as the 30 s averaged value at maximum recorded work rate. HR, respiratory exchange ratio (RER) and blood lactate were recorded every 2 min and at termination of the fitness test to validate $\dot{V}$O_2_peak criteria being reached. The test result was considered valid if heart rate exceeded 90% of age predicted maximum, RER > 1 and blood lactate concentration was greater than 8 mM at the end of the test (Milani et al., 2006; Poole et al., 2008; Horton et al., 2011).

#### Cognitive Data Scoring

Cognitive data were scored by hand by the same researcher, the Conners Continuous Performance test is computer based and scored automatically. Demeaned scores were calculated for correlation analysis with $\dot{V}$O_2_peak and MR data.

#### Image Analysis

Physiological noise correction was carried out using a modified RETROICOR (Glover et al., 2000) to remove cardiac and respiratory noise components from the data. ASL data were motion corrected using MCFLIRT within FSL (FMRIB’s Software Library;^1^ Jenkinson et al., 2002, RRID:SCR_002823). The CBF timeseries for the normocapnia and hypercapnia scans were corrected for coil sensitivity inhomogeneities using the minimal contrast images (Wang et al., 2005). Average difference images were obtained for each inversion time from tag-control subtraction of the CBF time series (Lu et al., 2006) and a perfusion map was created from all inversion times using a two-compartment CBF kinetic model implemented using the BASIL toolkit of FSL (Chappell et al., 2010). Within this framework, perfusion maps were converted to ml/100 g/min using the CSF signal as a reference to estimate the fully relaxed magnetization of water in blood (Chalela et al., 2000). The mean resting CBF for each subject was calculated by averaging the CBF time series over all voxels within the masked gray matter image (see below). CVR was calculated by dividing the percentage change in CBF from rest during hypercapnia by the mmHg change in P_ET_CO_2_ increase during hypercapnia.

#### Gray Matter Volume

T1-weighted structural data were analyzed with FSL-VBM (Good et al., 2001; Douaud et al., 2007).^2^ Structural images were brain extracted and segmented into gray matter, white matter and CSF before being registered to the MNI 152 standard space template using non-linear registration (Andersson et al., 2007). The resulting images were averaged and flipped along the x-axis to create a left-right symmetric, group-specific GM template. Second, all native GM images were non-linearly registered to this study-specific template and modulated to correct for local expansion (or contraction) due to the non-linear component of the spatial transformation. The modulated GM images were then smoothed with an isotropic Gaussian kernel with a sigma of 3 mm. Finally, voxel-wise GLM was applied using permutation-based non-parametric testing, correcting for multiple comparisons across space to investigate whether GM volume and $\dot{V}$O_2_peak were correlated.

#### ROI Analysis of Whole Gray Matter CBF and CVR

Individual subject gray matter masks were created using FMRIB’s Automated Segmentation Tool (FAST) and thresholded to include voxels with >50% gray matter probability. ROI analysis of the relationship between gray matter CBF at rest, CVR and $\dot{V}$O_2_peak was carried out using correlation analysis and permutation testing (100,000 permutations per comparison) in Matlab (Mathworks Inc., MA, United States). Permutation testing was used as an alternative to Bonferroni correction for multiple comparisons which is too conservative when potentially related variables are tested. This approach was also applied to examine the associations between gray matter CBF and CVR, and cognitive test scores.

#### Voxelwise Analysis of Gray Matter CBF and CVR

Follow up voxelwise analysis was conducted using FSL’s Randomize tool^3^ (Winkler et al., 2014) to examine the voxelwise correlation between (i) CBF and $\dot{V}$O_2_peak and (ii) CVR and $\dot{V}$O_2_peak. The individual subject masks described in the ROI Analysis section were used to confine this analysis to global gray matter only. Randomize is a permutation testing method that uses threshold free cluster enhancement (TFCE) (Smith and Nichols, 2009) to correct for multiple comparisons across voxels. Significance was set at *p* < 0.05 (FWE corrected).

## Results

All subjects completed the fitness test. Out of the 20 subjects 17 achieved an RER > 1.1 and 3 achieved RER > 1. 17 subjects exceeded the 90% HR maximum threshold and achieved a lactate peak >9, the remaining 3 exceeded 80% of HR maximum and achieved a lactate peak > 6. As this was a $\dot{V}$O_2_peak test all subjects satisfied test criteria by working to volitional exhaustion. Based on typical $\dot{V}$O_2_max criteria, 17 subjects fulfilled criteria for a maximal exercise test (Wilkinson et al., 2009; Dupuy et al., 2015). No adverse effects due to elevated CO_2_ inhalation during the MRI scans were reported in this sample. See Table 2 for group demographic and physiological data.

**TABLE 2:** Group characteristics and fitness test outcomes including $\dot{V}$O_2_peak and secondary validation criteria.

Characteristics	Mean (sd)
Sex (11 female, 9 male)	–
Age (years)	25 (4.6)
Weight (kg)	69.1 (8.8)
Height (cm)	173 (7)
BMI	23 (2.1)
$\dot{V}$O_2_peak (L/min)	2.9 (0.6)
$\dot{V}$O_2_peak (L/kg/min)	41.2 (8)
Baseline lactate mmol/L	0.96 (0.3)
Post-exercise lactate	9.5 (2)
Baseline HR	79.2 (18.8)
Peak HR	185.4 (9.8)
Baseline BP (pre- $\dot{V}$O_2_peak)	119/68
Peak RER	1.1 (0.04)
Maximum Work Rate (watts)	205 (40)
Baseline Borg (legs)	0.08 (0.2)
Peak Borg (legs)	8.5 (1.7)
Baseline Borg (breathing)	0.08 (0.3)
Peak Borg (breathing)	7.55 (2)
Baseline ETCO_2_	36.6 (3)
Hypercapnia ETCO_2_	44.2 (3.8)
ETCO_2_ Increase during hypercapnia	7.8 (1.4)

*ETCO_2_ values represent values recorded during the ASL scan.*

### Correlations Between CBF, CVR, and $\dot{V}$O_2_peak

#### ROI Analysis

**FIGURE 1:**
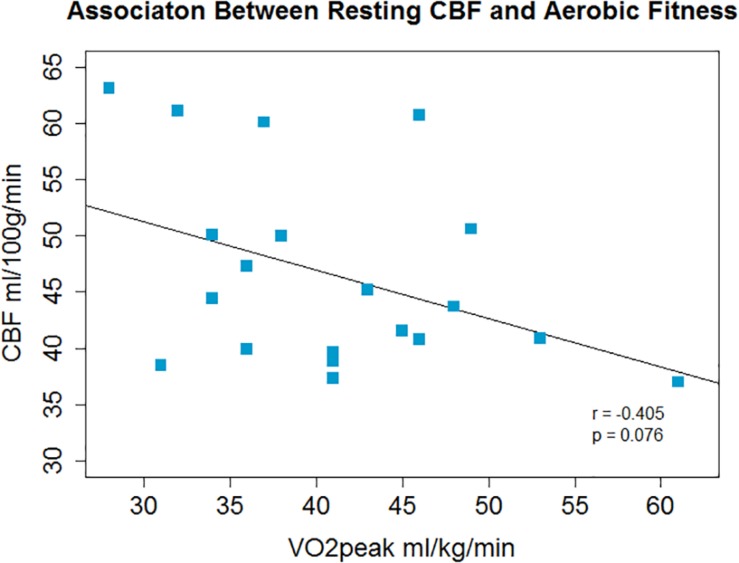
A non-significant inverse association between aerobic fitness and whole brain gray matter CBF was observed.

**FIGURE 2:**
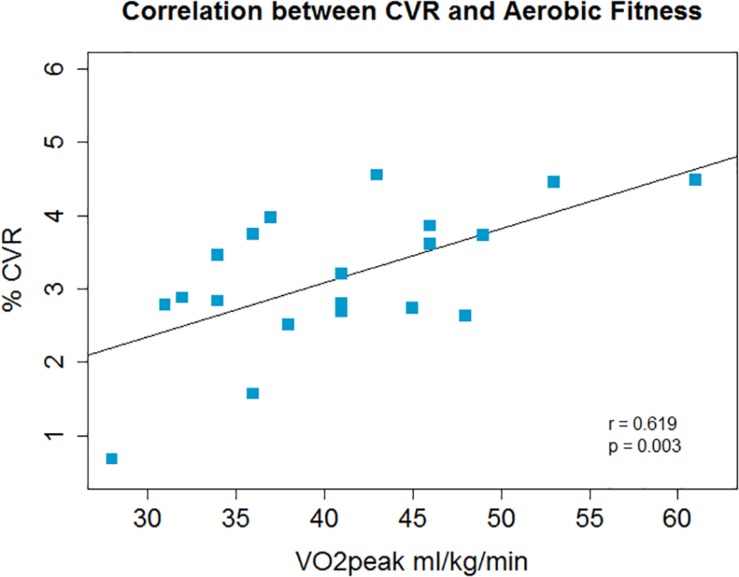
Across the group whole brain gray matter CVR and aerobic fitness were positively correlated.

There was a non-significant inverse association between $\dot{V}$O_2_peak and resting CBF in gray matter; *r* = −0.4, *p* = 0.08, *p*′ = 0.16 (Figure 1) and a significant positive correlation between $\dot{V}$O_2_peak and CVR; *r* = 0.62, *p* = 0.003, *p*′ = 0.006 (Figure 2).

Follow-up exploratory analysis showed that baseline P_ET_CO_2_ was not associated with $\dot{V}$O_2_peak; *r* = −0.05, *p* = 0.8 (Supplementary Figure S2), nor was the P_ET_CO_2_ change during hypercapnia associated with $\dot{V}$O_2_peak; *r* = 0.26, *p* = 0.27.

A supplementary power analysis to aid in the design of future studies in presented as Supplementary Table S3.

Group average gray matter CBF maps during rest and hypercapnia are shown in Supplementary Figure S5 for reference to demonstrate the CBF increase during hypercapnia.

#### Voxel-Wise Analysis

**FIGURE 3:**
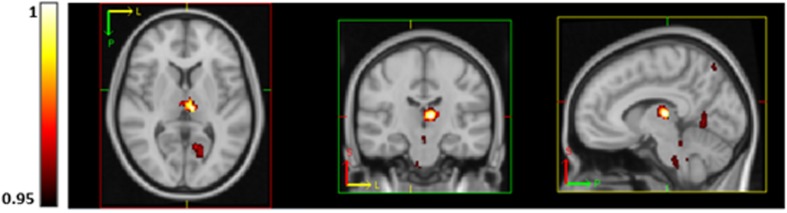
Regions of significantly lower CBF (using TFCE thresholding and FWE corrected) in subjects with higher aerobic fitness at rest in the thalamus, brainstem, precuneus, visual cortex (V1) and lingual gyrus. *P*-values are displayed as 1-p where a value of 1 is most significant.

Voxel-wise analysis revealed a significant inverse association between $\dot{V}$O_2_peak and resting CBF in the left and right thalamus, brainstem, right lateral occipital cortex, left intra-calcarine cortex and cerebellum (Figure 3). No regions were significantly positively correlated with $\dot{V}$O_2_peak. Voxel-wise CVR did not correlate with $\dot{V}$O_2_peak.

#### Cognitive Performance, Gray Matter Volume, and $\dot{V}$O_2_peak

Finally, correlation analysis did not reveal any significant associations between $\dot{V}$O_2_peak and cognitive performance, or cognitive performance and cerebrovascular measures. Mean scores and correlations are shown in Supplementary Tables S1, S2. VBM analysis of gray matter volume did not show a trend or association with $\dot{V}$O_2_peak.

## Discussion

In this study, we examined the association between aerobic fitness and resting CBF and CVR using multiple inversion time PASL. This is the first study to report associations between aerobic fitness, CBF-based CVR, as opposed to BOLD-based CVR, and whole-brain gray matter CBF in young adults using ASL. Across the group, higher $\dot{V}$O_2_peak was associated with lower regional resting CBF and greater global gray matter CVR to CO_2_. $\dot{V}$O_2_peak was not associated with cognitive task performance or gray matter volume in this cohort.

### $\dot{V}$O_2_peak, CBF and CVR

The inverse correlation between CBF and $\dot{V}$O_2_peak was not expected, as the opposite has been found in children (Chaddock-Heyman et al., 2016), young adults (Bailey et al., 2013) and older adults (Ainslie et al., 2008; Brown et al., 2010; Bailey et al., 2013; Thomas et al., 2013; Zimmerman et al., 2014). However, with the exception of Thomas et al. (2013); Zimmerman et al. (2014), and Chaddock-Heyman et al. (2016), previous studies have used TCD ultrasound. TCD measures blood flow velocity, not flow, as TCD does not measure vessel diameter which would be required to calculate flow using this method. In contrast, ASL measures CBF directly through the labeling of inflowing water to tissue. Therefore, results may differ as they are measuring different vascular parameters. Recent efforts to characterize differences between MRI and TCD measures of CVR are valuable and will allow the methods to be used as complementary techniques given that TCD can be conducted under conditions not suited to MRI. For example, Burley et al. (2017) reported a positive correlation between VO_2_max and CVR using both BOLD fMRI and TCD suggesting that the methods are not necessarily contradictory (Bailey et al., 2013; Thomas et al., 2013) and could be used to investigate brain function within groups at rest and during exercise. The main limitations of TCD are that it lacks the spatial resolution and the ability to measure tissue perfusion that is afforded by quantitative ASL MRI.

Most existing MRI studies investigated groups undergoing neurodevelopment (Chaddock-Heyman et al., 2016) or older adults (Thomas et al., 2014; Zimmerman et al., 2014) where the mechanistic relationships with aerobic fitness may be different to adults not in stages of either development or decline which may explain why results are not consistent across studies with different age groups. Further evidence for differential effects of aerobic fitness with age comes from a recent structural MRI study by Williams et al. (2017) which reported a positive correlation between cortical thickness and $\dot{V}$O_2_peak in older adults but an inverse correlation between these measures in young adults. CBF quantification in older adults must also be interpreted with caution where partial volume effects have not been controlled for. Coarse spatial resolution of ASL combined with cortical atrophy and a lack of correction for partial volume errors, plus delayed bolus arrival times may mean that perfusion is mis-estimated in the aging brain.

In the present study, $\dot{V}$O_2_peak showed a non-significant inverse correlation with CBF averaged across the whole of gray matter. Statistical significance of an inverse correlation between CBF and $\dot{V}$O_2_peak was localized to regions of the thalamus, brainstem, visual cortex, cerebellum and precuneus. Regional variation in contrast-to-noise may explain why CBF in these specific regions correlated with fitness in this sample. Even though the same direction of effect was observed in both the ROI and voxel-wise analyses, the results should be interpreted with caution given the small sample size of the present study. Although the direction of the relationship between $\dot{V}$O_2_peak and baseline CBF was the opposite to that predicted at the outset of the study, the result is plausible in the light of a recent corroborative finding from our group in an independent cohort of young healthy volunteers. The study by Furby et al. (2019) was conducted using similar ASL MRI methods and demonstrated an inverse correlation between fitness and whole-brain averaged gray matter CBF. The interpretation of regional inverse correlations of baseline CBF with $\dot{V}$O_2_peak remains speculative in the present study. It is possible that sensory gating in relation to fitness, in association with physical training, may affect the tonic level of activity in the thalamus as there is evidence of altered sensorimotor gating in athletes (Hormigo et al., 2019). However, it must be noted that participants in the present study were not selected for their high level of athletic performance.

A study by Robertson et al. (2015) in stroke survivors, using single inversion time PCASL, similarly found an inverse correlation between precuneus CBF and aerobic fitness, but a positive correlation between thalamic and posterior cingulate CBF and aerobic fitness. Gauthier et al. (2015) found both positive and inverse correlations between CVR and aerobic fitness in older adults, and this, along with the Robertson et al. (2015) study adds to the evidence that aerobic fitness may have complex, regionally varying and age dependent effects on the brain. Although the direction of the correlation in stroke survivors in the thalamus and posterior cingulate cortex contradicts the current results, the current study and those of Robertson et al. (2015) and Gauthier et al. (2015) further suggest that aerobic fitness may exert localized effects on CBF (Thomas et al., 2013; Chaddock-Heyman et al., 2016). Given that participants in each of these cited studies have differed in age and health status, the fact that consistent findings have not been reported is unsurprising. The variance in the effects of aerobic fitness on brain function with age needs further investigation through interventions in young and older groups as there may be a cross-over effect whereby higher CBF in youth and older adulthood has different functional importance. The current study consists of a normal sample of young adults who varied in fitness level, but not to the extent that a cross-sectional sample comprised of athletes and sedentary individuals would. However, our sample had a mean $\dot{V}$O_2_peak of 41 ml/kg/min, standard deviation 8, similar to Hwang et al. (2017) who reported an average $\dot{V}$O_2_max of 37.8, standard deviation 9.6 in a sample of 87 participants and also found a positive correlation between aerobic fitness and CVR in the MCA. The range of fitness levels within the study sample is likely to dictate the degree of observed fitness-related brain effects, especially in healthy groups. In fact, as well as differences in study design, the spread of fitness levels within studies may also explain the differences in the results of Barnes et al. (2013) and Bailey et al. (2013). Bailey et al. (2013) compared CVR between two groups, sedentary vs. trained, whereas Barnes et al. (2013) used a single group with a range of fitness levels who did not participate in a structured training regime.

We offer a neurobiological interpretation which potentially explains the negative correlation between CBF and $\dot{V}$O_2_peak in this study. However, we stress that we do not have further data to support this mechanistic interpretation instead it forms a hypothesis that requires further investigation. Regular exercise training which increases $\dot{V}$O_2_max, can result in increased tissue capacity to extract oxygen from blood in muscle (Kalliokoski et al., 2001; Ookawara et al., 2002), potentially as a result of greater blood vessel density which facilitates oxygen diffusion throughout the tissue. Similarly young animals subjected to wheel running for several weeks have shown increased angiogenesis, and therefore capillary density, in the brain compared with sedentary animals (Swain et al., 2003; Pereira et al., 2007). Should this also be the case in humans, greater capillary density would be associated with enhanced oxygen diffusivity into tissue (Germuska et al., 2019), and higher oxygen extraction fraction (OEF), implying that a lower CBF is needed to sustain a given cerebral rate of oxygen metabolism. In terms of oxygen metabolism, future studies examining the relationship between aerobic fitness, CBF and OEF are needed.

Animal models can shed light on the mechanisms underpinning the effects of fitness on the brain at the microscopic and biochemical level. In particular, such work has demonstrated increased mitochondrial biogenesis following exercise training, in regions including the brainstem, cerebellum and hippocampus (Steiner et al., 2011). Mitochondrial dysfunction plays a role in many diseases for which aerobic fitness has been shown to reduce the incidence, such as cardiovascular disease and neurodegenerative diseases such as dementia (Navarro et al., 2009; Bertram et al., 2016). Similarly, exercise may influence angiogenesis (Black et al., 1990) as discussed above, and synaptogenesis in the form of region-specific increases in presynaptic density in the dentate gyrus and CA3 region of the hippocampus as demonstrated in aged rats (Siette et al., 2013). Changes at the synaptic level could drive metabolic demand and it is important to understand these changes in humans to fully interpret functional MRI data. Recent advances in PET imaging now allow potential biomarkers of synaptic density to be measured, for example, synaptic vesicle glycoprotein 2A (SV2A) (Nabulsi et al., 2016) can be targeted with an SV2A radiotracer to quantify this index of synaptic density to be compared between individuals with different levels of aerobic fitness.

Upon neuronal activation, there is an increase in demand for energy; in the healthy brain this is met with an increase in CBF. The lower resting CBF observed in higher fitness individuals, although initially counterintuitive, is not problematic if there is an adequate vascular reserve to meet changes in energy demand. The data support this argument as a greater gray matter CVR to CO_2_ was observed in subjects with a higher $\dot{V}$O_2_peak. If aerobic fitness maintains CBF and oxygenation, both of which are affected by aging (Aanerud et al., 2012), then an increase in CBF, and potentially CVR, in the trained group, would be expected when comparing trained vs. sedentary older adults. However, if aerobic fitness also enhances oxygen extraction efficiency, in young people this so-called efficiency may be observed as a lower resting CBF if aerobic fitness does indeed increase the number of blood vessels and therefore CBV as outlined above. Greater vessel density would increase the capacity for oxygenation and oxygen diffusion gradient across the tissue. In addition, CVR and $\dot{V}$O_2_peak may then be positively correlated due to a higher vascular reserve, leading to a greater CBF response to CO_2_ as CBF increases well above what is required to respond to a stimulus (Fox and Raichle, 1986). Future work which quantifies OEF, CBF, and CVR in similar populations could provide the additional necessary information to understand cerebral vascular and metabolic function at different levels of aerobic fitness.

The ROI analysis showed a positive correlation between CVR and $\dot{V}$O_2_peak in global gray matter, however, no specific regions emerged in a follow-up voxel-wise analysis. CVR declines with age (De Vis et al., 2015; Peng et al., 2018) but in the literature, there are contradictory reports on the association between CVR and aerobic fitness (Bailey et al., 2013; Barnes et al., 2013; Thomas et al., 2013; Gauthier et al., 2015), as discussed in the introduction. The positive association between $\dot{V}$O_2_peak and CVR reported here is in line with the TCD findings by Bailey et al. (2013), whereby trained young adults had greater CVR than a sedentary comparison group. However, few studies have investigated the relationship between CVR and aerobic fitness in healthy young adults, therefore more work is needed to adequately understand this relationship. Overall results across studies using MRI and TCD to study CBF, flow velocity and CVR suggest that exercise training and aerobic fitness has complex and varying effects on brain regions with potential mechanistic differences across the lifespan. This complexity presents an important area of future research in different age groups, and a need to comprehensively map cerebrovascular function across the brain. Future studies using both TCD and ASL would also add important information on the central and vascular effects of aerobic fitness on CBF and the source of any differences between the two methods.

Finally, it should be noted that De Vis et al. (2015) showed that resting P_ET_CO_2_ accounted for age-related CBF differences almost entirely. In this study, there was a non-significant positive trend (*r* = 0.4, *p* = 0.06) between baseline P_ET_CO_2_ and CBF, but no strong associations between $\dot{V}$O_2_peak and baseline P_ET_CO_2_ (*r* = 0.05, *p* = 0.8) or the ΔP_ET_CO_2_ during hypercapnia (*r* = 0.26, *p* = 0.27, see Supplementary Figures S2–S4). This suggests a cerebrovascular effect of fitness on CVR rather than an effect driven by P_ET_CO_2_ differences.

### Cognitive Performance and Gray Matter Volume

Cognitive performance scores were similar across the group and there were no strong associations with $\dot{V}$O_2_peak nor with CBF or CVR (see Supplementary Tables S1, S2). It is possible that cognitive differences in young adults may only be visible at more extreme ends of the fitness spectrum than sampled in this study. It is also possible that more sensitive cognitive tests are required to detect differences among high functioning groups separated only by fitness level.

The cognitive reserve hypothesis (Chodzko-Zajko and Moore, 1994) states that higher fitness in older age offsets age-related decline in cerebral circulation, enhancing oxygen delivery to support neural demand. More recently, Etnier et al. (2006) conducted a meta-regression on studies examining the effects of fitness on cognitive function. In summary, the authors did not find strong support for a beneficial effect of fitness in any age group once moderator variables were considered, and in fact found a negative association between fitness and cognition in studies using a pre-post design. Only a very small number of studies report a beneficial effect of fitness or physical activity on select cognitive domains in young people (Themanson et al., 2009; Baym et al., 2011; Stroth et al., 2016) and other studies report significant effects in older but not young adults (Hayes et al., 2014b). In summary, more work is needed in young adults to determine neurophysiological effects of aerobic fitness and their functional relevance in terms of health and cognition.

Aerobic fitness is believed to reduce age-related atrophy, mainly in the hippocampus (Erickson et al., 2011; Firth et al., 2018). In this study the focus was on cerebrovascular function, not brain structure, however, if structural differences were present, this would affect CBF and CVR. The VBM analysis showed that gray matter global volume differences due to fitness were not present. As this group was young and healthy, this finding is not wholly surprising, and suggests that volumetric differences only become apparent in later life, adding to our knowledge of the lifelong effects of aerobic fitness.

### Limitations

Hemoglobin (Hb) levels were not measured. Hb is responsible for transport of O_2_ to tissue. The concentration of Hb in blood affects exercise performance; lower Hb means that blood can carry less oxygen (Calbet et al., 2006) and therefore muscle function is impaired. In addition, Hb affects perfusion estimates as there is an inverse relationship between [Hb] and the longitudinal relaxation time (T_1_) of blood. Brain capillary [Hb] cannot be measured directly *in vivo*, however, peripheral capillary samples may provide an indication of Hb differences between subjects or groups which could help to explain biological mechanisms driving CBF differences.

Second, investigations were restricted to gray matter. Due to the limited SNR of ASL, reliable quantification of CBF and CVR in white matter is difficult as CBF is much lower than in gray matter. However, adequate blood supply and energy metabolism is necessary for all round brain health, and aging is associated with damage to white matter microstructure and reductions in myelination (Gunning-Dixon et al., 2009). Therefore, improvement of methods to study the WM vasculature are also needed to understand the global effects of fitness.

The voxelwise and ROI analyses did not show the same statistical significances. While the voxelwise analysis of CBF revealed regions of significantly lower CBF with fitness, the ROI analysis showed a non-significant trend in the same direction for global gray matter. The ROI analysis of CVR, however, showed a moderate (0.6) significant correlation with fitness while the voxelwise analysis did not reach statistical significance. ASL is an intrinsically low SNR technique compared to BOLD fMRI. Although ASL offers significant benefits in terms of quantification of physiological parameters, the SNR may have prevented detection of greater voxelwise associations with $\dot{V}$O_2_peak. An alternative possibility is that CBF is more heterogeneous throughout the brain than CVR which is more affected by low SNR; therefore greater averaging of data in the ROI analysis allowed CVR differences to be detected.

Lastly, there are known hormonal effects on exercise test performance (de Jonge, 2003) and CBF (Brackley et al., 1999; Duckles and Krause, 2007). In the present study, the resting CBF difference was not significant between males and females; *t*(18) = −0.119, *p* = 0.734 but controlling for menstrual cycle phase, which was not done here, could reduce variability within cohorts. This is a preferable option to limiting studies to males only as hormonal differences may play a role in the acute response to exercise and possibly in mediating the effects of fitness on brain health.

### Summary and Future Directions

The results of this study suggest that aerobic fitness is associated with lower CBF and greater CVR in young, healthy adults, however, the modest effects observed need replication in larger samples. We currently know very little about the functional relevance of CBF or CVR differences in young adults, and how the observed neurophysiological effects of physical training differ from those observed in older adults. With recent advances in quantitative MRI techniques, non-invasive mapping of multiple indices of cerebrovascular function, e.g., CBF, CVR, CBV OEF and the cerebral metabolic rate of oxygen consumption is possible within a single scan session (Bulte et al., 2012; Gauthier et al., 2012; Gauthier and Hoge, 2013; Wise et al., 2013). Notably, regional CBV quantification would bring us a step closer to comparisons with experimental data on angiogenesis and capillary density following exercise.

Application of these techniques to study brain function in both young and older trained and sedentary adults will provide information necessary to move forward in developing exercise training protocols to increase the adoption of fitness training as a preventative health tool.

Research over the next decade should also work to establish whether regular exercise regardless of intensity level delivers brain benefits, or whether there is an aerobic fitness threshold, above which benefits such as maintained CBF with aging, are observed. Answering this question will guide optimal exercise dose recommendations and interventional studies.

## Author’s Note

This manuscript can be found as a preprint on BioRxiv (Foster et al., 2019) at: https://www.biorxiv.org/content/10.1101/539072v1. This work first appeared in the lead author’s thesis (Foster, 2017; Chapter 4) which can be accessed here: http://orca.cf.ac.uk/109647/.

## Data Availability Statement

The datasets generated for this study are available on request to the corresponding author.

## Ethics Statement

The study was approved by the Cardiff University School of Psychology Research Ethics Committee and performed in accordance with the guidelines stated in the Cardiff University Research Framework (version 4.0, 2010). Informed written consent was obtained from all subjects.

## Author Contributions

CF conceived and designed the study with input from RW. CF and JS coordinated the project and collected the data with assistance from DH. CF and RW analyzed the data. CF prepared the manuscript with RW providing input and interpretation of results as well as reviewing and editing the final manuscript. VT supervised CF throughout the project. All authors approved the manuscript before submission.

## Conflict of Interest

The authors declare that the research was conducted in the absence of any commercial or financial relationships that could be construed as a potential conflict of interest.

## Footnotes

1 www.fmrib.ox.ac.uk/fsl

2 http://fsl.fmrib.ox.ac.uk/fsl/fslwiki/FSLVBM

3 http://www.fmrib.ox.ac.uk/fsl/randomise/index.html
