# Supplementary material for: Assessment of the Effects of Aerobic Fitness on Cerebrovascular Function in Young Adults Using Multiple Inversion Time Arterial Spin Labeling MRI
Source: Front Physiol. 2020 Apr 21;11:360. doi: 10.3389/fphys.2020.00360 (PMC7187806; doi:10.3389/fphys.2020.00360)
Supplement: Supplementary file 1 [file Table_1.docx]

Supplementary Data


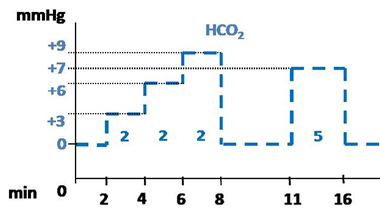


Supplementary Figure 1. Protocol for the pre-MRI respiratory modulation test session. Heart rate and oxygen saturation were monitored and recorded throughout the session. Figure adapted from Merola, (2016). HCO2 = End-tidal CO2 during hypercapnia.


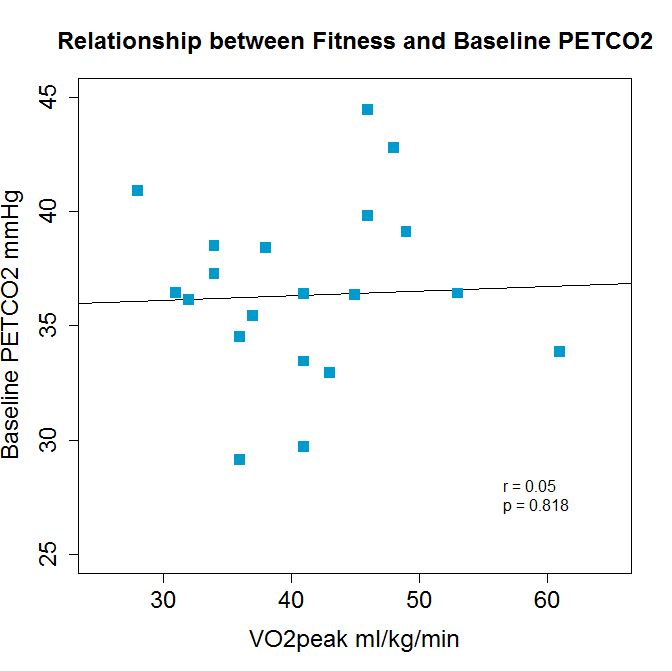


Supplementary Figure 2. Aerobic fitness was not a predictor of baseline P_ET_CO_2_.


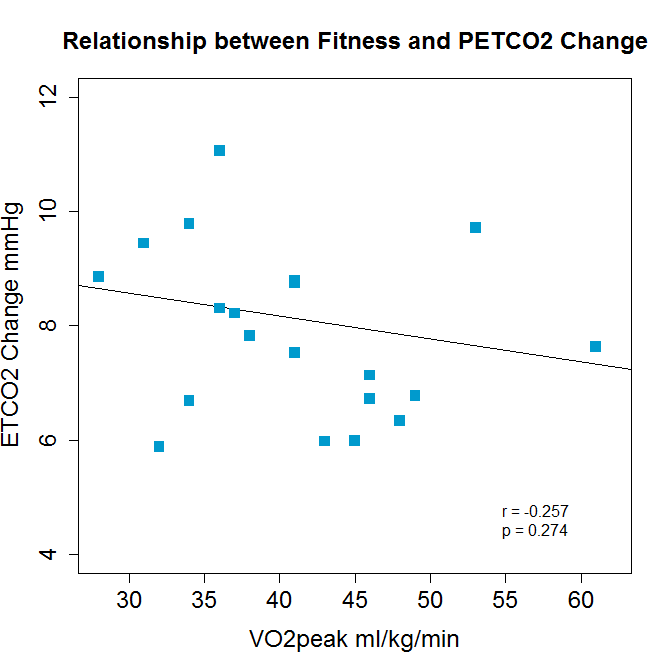


Supplementary Figure 3. Aerobic fitness was not a predictor of ΔP_ET_CO_2_ during hypercapnia.


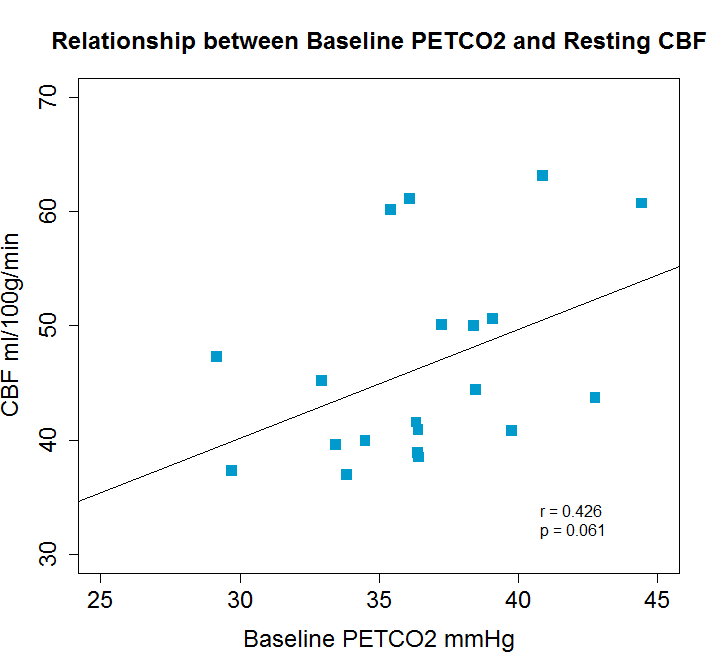


Supplementary Figure 4. There was a non-significant positive association between resting CBF and baseline P_ET_CO_2_.


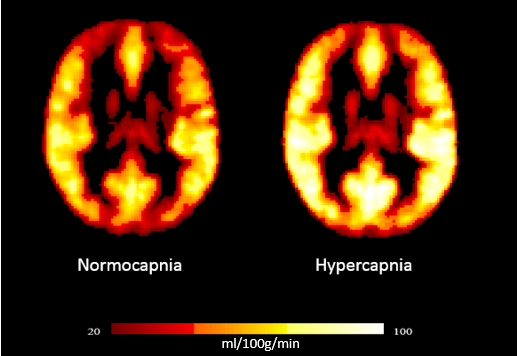


Supplementary Figure 5 Average grey matter CBF maps (n=20 participants) during rest and hypercapnia.

| Cognitive Test | Mean (sd) |
| --- | --- |
| Conners Continuous  Performance (d’) | 43.6 (7.6) |
| SDMT | 62 (10) |
| Trail Making B | 51.3 (28.5) (s) |
| SCOLP (max 100) | 81 (17) |
| Digit Span (LDSF) | 6.8 (1.4) |
| Letter Fluency | 45.1 (8.6) |
| HADS A | 3.75 (2.2) |
| HADS D | 0.7 (0.9) |

Supplementary Table 1. Group average and standard deviation for each cognitive test.

| Correlation Analysis with Cognitive Tasks | | | |
| --- | --- | --- | --- |
| Cognitive Test | **CBF** | **CVR** | $\dot{\boldsymbol{V}}$**O2peak** |
| SCOLP | r = 0.06  p = 0.80, p’ = 1.00 | r = 0.26,  p = 0.27, p’ = 0.832 | r = -0.02,  p = 0.95, p’ = 1.00 |
| Digit Span | r = 0.04,  p = 0.88, p’ = 1.00 | r = -0.07,  p = 0.76, p’ = 1.00 | r = -0.46,  p = 0.04, p’ = 0.233 |
| Letter Fluency | r = -0.16,  p = 0.50, p’ = 0.982 | r = 0.47,  p = 0.04, p’ = 0.205 | r = 0.27,  p = 0.25, p’ = 0.808 |
| Conners Continuous Performance d’ | r = -0.31,  p = 0.18, p’ = 0.675 | r = 0.38,  p = 0.10, p’ = 0.46 | r = 0.24,  p = 0.31, p’ = 0.878 |
| Trail Making B | r = -0.02,  p = 0.93, p’ = 1.00 | r = 0.01,  p = 0.97, p’ = 1.00 | r = 0.20,  p = 0.40, p’ = 0.947 |
| SDMT | r = 0.24,  p = 0.32, p’ = 0.889 | r = 0.08,  p = 0.75, p’ = 0.999 | r = -0.14,  p = 0.56, p’ = 0.991 |

Supplementary Table 2. Correlations between cognitive task performance and fitness as well as resting CBF and CVR. p' = corrected p value.

| Power Analysis | | | |
| --- | --- | --- | --- |
| Post-Intervention Correlations with $\dot{\boldsymbol{V}}$O_2_peak | Effect Size (r) | Power | Sample Size required for power of 0.8 at p<0.05 |
| Resting CBF | 0.405 | 0.45 | 44 |
| CVR | 0.619 | 0.91 | 15 |

Supplementary Table 3 Power analysis conducted post-hoc to determine power and sample sizes for future studies. All calculated using the correlation coefficient (r) in G*Power (v3.1.9.2) using grey matter averaged estimates of CBF and CVR.
